# Laugh with Me: A Case Study of Easing a Caregiver’s Burden through Music

**DOI:** 10.1002/alz.092462

**Published:** 2025-01-09

**Authors:** Kendra Ray, Marion Kaiser, Mary S Mittelman

**Affiliations:** ^1^ New York University School of Medicine, New York, NY USA; ^2^ Menorah Center for Rehabilitation and Nursing Care, Brooklyn, NY USA; ^3^ NYU School of Medicine, New York, NY USA

## Abstract

**Background:**

In the United States, approximately 2 million caregivers of people with Alzheimer’s disease or related dementia (ADRD) are Latin American. As many of these caregivers are family or friends, most are not equipped with the tools necessary to address common behaviors associated with ADRD. Music‐based interventions are an effective and accessible tool to assist caregivers in addressing these persistent behaviors. Here, we provide an example of a Latina caregiver for her mother who used music to help relieve symptoms of ADRD.

**Method:**

Carmen (pseudonym) attended a 6‐week music‐based, psychoeducational intervention explicitly created for caregivers of people with ADRD via Zoom for 1 hour. She incorporated music specific to her culture with songs like “Metete Teté” during the six weeks. The Neuropsychiatric Inventory (NPI‐Q) and Short‐Form Zarit Burden Interview were completed at pretest and posttest. A rewrite of “*Stand by Me”* occurred in week six. A comparison of scores was documented, and then a percentage was calculated to determine changes between the pretest and posttest. Informed by grounded theory, a lyric analysis of the rewrite of “Stand by Me” involved creating codes for each textual idea, which led to developing categories. Themes were then revealed based on the organization of codes and categories.

**Result:**

Carmen’s burden decreased by 47% between pretest and posttest. Comparatively, there was a 77% decrease in the severity of persistent behaviors in her mom. Additionally, Carmen’s distress was reduced by 87% following the 6‐week intervention. Carmen reported that agitation remained at posttest, which contributed to mild caregiver distress. In her rewrite of the lyrics, Carmen retitled the song, “Laugh with Me.” Four themes revealed include ‘*changes in the environment during sunset’, ‘agitation appears’, ‘no stress, no fear’, ‘laughter*, *and togetherness as the solution’*.

**Conclusion:**

This case example demonstrated the use of a music‐based intervention facilitated by a Latina caregiver, resulting in reductions in caregiver burden, caregiver distress, and neuropsychiatric symptoms in her mom. Using a case example allowed a closer analysis of NPI‐Q posttest scores, which revealed ongoing agitation. Comparatively, the lyric analysis described how agitation arises and potential caregiving techniques used to resolve agitation.

